# A Window into the Brain Demonstrates the Importance of Astrocytes

**DOI:** 10.1371/journal.pbio.0020115

**Published:** 2004-04-13

Did you ever wish you could peek inside someone's brain and see what was going on in there? In research reported in this issue of *PLoS Biology*, Hajime Hirase and his colleagues at Rutgers University have done just that by focusing their microscope on the brains of living rats in order to examine how certain cells called astrocytes function in vivo.[Fig pbio-0020115-g001]

**Figure pbio-0020115-g001:**
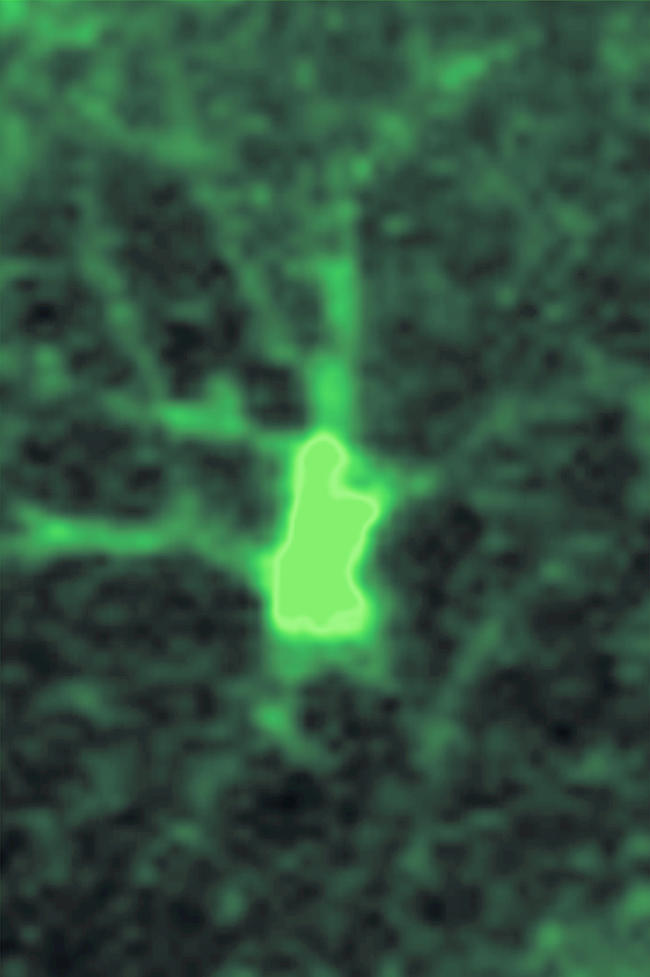
Astrocyte in the cerebral cortex

In the longstanding quest to understand how the brain works, scientists have focused on neurons. Neurons conduct action potentials, electrical signals that transmit information in the nervous system. But the brain also contains several other types of cells called glia. (*Glia* is derived from the Latin for “glue”; these cells were thought to “hold it all together.”) One type of glial cell, the astrocyte (named for its starlike shape), is the most populous cell in the brain and forms an intimate association with neurons and their synapses. It was thought that these cells played a supporting role in the brain, ensuring the proper chemical environment for synapses.

Recent research, however, has suggested that astrocytes and other glial cells may play a more significant role. When examining astrocytes cultured in the lab, scientists have observed behavior suggesting that astrocytes can communicate with neurons. Though astrocytes cannot propagate electrical signals like neurons do, they can sense the transmission of such signals at the synapse between two neurons. Furthermore, astrocytes are able to propagate a different kind of signal, a chemical signal based on the release of calcium ions. Calcium signaling is a mechanism of chemical signaling that has been observed in many other cell types. The exact properties of neuron–astrocyte communication, however, are not clear because different preparations of these tissues have yielded different results. It has also not been established that this type of communication occurs in the living brain.

To explore such questions, Hirase and colleagues have taken the next step by investigating the calcium signaling properties of astrocytes in the brains of living rats. To accomplish this feat, the researchers used a combination of two technologies. They monitored calcium signaling using a fluorescent dye called Fluo-4, which fluoresces in response to calcium ions. Then they used a special type of microscope called a two-photon laser scanning microscope to visualize the dye. Since this type of microscope uses a lower energy laser, it can image the dye in living tissue without causing harm.

The researchers applied the dye to the brains of anesthetized rats, washed out the excess dye that had not penetrated into cells, and then imaged the tissue under the microscope. They first confirmed that they indeed were examining astrocytes and noticed that cells displayed a moderate level of baseline calcium signaling activity. They then used a drug called bicuculline to stimulate neurons and observed a significant increase in the calcium signaling activity of the astrocytes. Because bicuculline only affects neurons, this implies that the astrocytes are responding to the activity of the neurons. The researchers also found that neighboring astrocytes often also displayed coordinated calcium signaling activity, suggesting that the communication among astrocytes is facilitated by increased neuronal activity.

This research confirms the complexity of astrocyte signaling functions in the living brain and demonstrates that astrocytes play far more than a supporting role in brain function. It also establishes an important experimental system for scientists seeking to understand how these distinct elements of the brain—neurons and astrocytes—work together. Though this research makes it clear that signaling exists both among astrocytes and between neurons and astrocytes, scientists have yet to understand the effect of this signaling. Some possibilities include regulation of synapse formation, modification of synaptic strength, or more complicated roles in information processing resulting from the coordination of neuronal activity. Future research using this and other systems will help reveal these functions.

